# Characteristics of the Follicular Fluid Extracellular Vesicle Molecular Profile in Women in Different Age Groups in ART Programs

**DOI:** 10.3390/life14050541

**Published:** 2024-04-24

**Authors:** Anastasia Sysoeva, Zumriyat Akhmedova, Oksana Nepsha, Natalya Makarova, Denis Silachev, Yulia Shevtsova, Kirill Goryunov, Victoria Karyagina, Anna Bugrova, Natalya Starodubtseva, Anastasia Novoselova, Vitaliy Chagovets, Elena Kalinina

**Affiliations:** 1 V.I. Kulakov National Medical Research Center for Obstetrics Gynecology and Perinatology, Ministry of Healthcare of Russian Federation, 117997 Moscow, Russia; a_sysoeva@oparina4.ru (A.S.); b_zingerenko@oparina4.ru (Z.A.); o_nepsha@oparina4.ru (O.N.); np_makarova@oparina4.ru (N.M.); u_shevtsova@oparina4.ru (Y.S.); k_gorunov@oparina4.ru (K.G.); v_karyagina@oparina4.ru (V.K.); a_bugrova@oparina4.ru (A.B.); n_starodubtseva@oparina4.ru (N.S.); a_novoselova@oparina4.ru (A.N.); v_chagovets@oparina4.ru (V.C.); e_kalinina@oparina4.ru (E.K.); 2 A.N. Belozersky Institute of Physico-Chemical Biology, Lomonosov Moscow State University, 119992 Moscow, Russia; 3 Emanuel Institute of Biochemical Physics, Russian Academy of Sciences, 119334 Moscow, Russia; 4 Moscow Institute of Physics and Technology, 141700 Moscow, Russia

**Keywords:** extracellular vesicles, exosomes, follicular fluid, sperm, aging, progesterone, metabolome, proteome

## Abstract

The aim of this study was to investigate the molecular composition of follicular fluid (FF) extracellular vesicles (EVs) in women of different reproductive ages and its possible relationship to sperm fertilizing ability. FF EVs were obtained by differential centrifugation. The concentration and size distribution of FF EVs were analyzed by nanoparticle tracking analysis. The lipidome and proteome were analyzed by liquid chromatography–mass spectrometry. The isolated FF EVs had a variety of shapes and sizes; their concentration and size distribution did not differ significantly between the age groups. In women younger than 35 years, the concentration of vesicular progesterone was 6.6 times higher than in women older than 35 years, and the total levels of the main lipid classes were increased in younger women. A proteomic analysis revealed that not only FF EV-specific proteins, but also proteins involved in sperm activation were present. New data were obtained on the composition of FF EVs, confirming their importance as molecular indicators of age-related changes in the female reproductive system. In addition, these results shed light on the possible interaction between the FF EVs of women in different age groups and male germ cells. Therefore, studying the transcriptomic and metabolomic profile of FF EVs may be a crucial approach to evaluate the efficacy of ART.

## 1. Introduction

This work is part of a large-scale study on the influence and interaction of follicular fluid extracellular vesicles (FF EVs) with human sperm in the female reproductive tract during fertilization. In our previous work, we were the first to demonstrate the peculiarities of the binding of FF EVs from women of different ages to the sperm membrane and we also demonstrated a significant improvement in the characteristics of motility, hyperactivation and membrane state changes necessary for fertilization [1].

Extracellular vesicles are bilayered phospholipid membrane structures secreted by all types of cells and containing various bioactive molecules (proteins, RNA, mRNA, microRNA and lipids) [2,3]. According to the recommendations of the International Society for Extracellular Vesicles (ISEV) [4] 2018, there are several criteria for classifying EVs: size, density and biochemical composition [5]. EVs are divided into the following types: exosomes (30–100 nm), ectosomes (100–350 nm), microvesicles (100 nm to 1 µm) and apoptotic bodies (1–5 µm) [4,5,6]. A number of studies showed that EVs play an important role in physiological processes such as cell proliferation and differentiation, cell signaling, immune responses, inflammatory processes, as well as gametogenesis, fertilization, implantation and embryonic development [7,8]. FF EVs stand out in a special way from the vesicles of other biological fluids; not only has their role been described in oogenesis in many mammalian species, but also, in 2019, it was suggested for the first time that EVs are involved in the activation of sperm in the reproductive tract [9].

Age-related changes in the body lead to a progressive deterioration in the physiological functions of tissues and organs, gradually reducing the quality of life [5,6]. It is believed that the reproductive system, especially the ovaries, show signs of aging at an earlier stage of life (around 35 years) compared to other internal organs. At the same time, their functional activity, which is necessary for maintaining the quality of the oocytes, decreases, and apoptotic and proliferative processes begin to predominate and prepare the body for menopause [10,11]. Numerous studies showed that fertility declines with increasing age, which is due to a decrease in the quantity and quality of ovarian reserve as well as to an increased risk of miscarriage, pregnancy loss, poor response to ovarian stimulation and oocyte abnormalities such as chromosomal aneuploidies [11,12]. Therefore, the pregnancy rates are lower for women of advanced reproductive age than for women under 35 years of age, so that a significant proportion of patients with unsuccessful outcomes of assisted reproductive technology (ART) programs are women of advanced reproductive age [11]. There are several key features of aging, including cellular aging and changes in intercellular communication [1,5,13]. Due to their crucial role in intercellular interactions, FF EVs are necessary not only for maintaining ovarian follicle homeostasis [6], but also as mediators between the oocyte and sperm in the oviduct to trigger and regulate their fertilization ability. This study provides new insights into the molecular composition of FF EVs, which may help to understand the causes of infertility of unknown origin as well as clarify the role of FF EVs as activators of male germ cells and new biomarkers in the aging process to improve the treatment of infertility.

## 2. Materials and Methods

### 2.1. Biological Material

The biological material was obtained from the V.I. Kulakov National Medical Research Center for Obstetrics Gynecology and Perinatology. The follicular fluid was obtained from donors who underwent examination in the department of assistive technologies for the treatment of infertility in the period from September 2021 to December 2022, in accordance with the Order of the Ministry of Health of the Russian Federation No. 107n “On the procedure for the use of assisted reproductive technologies, contraindications and limitations to their use” of 2013.

FF was obtained from young patients (*n* = 15) <35 y.o. (20–33 years), without aggravated medical history, with normal body mass index (BMI) and at their first IVF attempt and from women >35 y.o. (41–47 years) (*n* = 15) with recurrent implantation failure. The main factors of infertility in the >35 y.o. group were the tubal–peritoneal factor and repeated unsuccessful IVF attempts. The mean value of BMI in young patients was 22.7 ± 2.0 kg/m^2^, and that in the advanced-maternal-age (AMA) group was 23.2 ± 1.4 kg/m^2^, which are normal values. Ovarian stimulation was the same in all women and was performed with an initial dose of 150–200 IU of recombinant follicle-stimulating hormone (GONAL-F, Merck Serono, Roma, Italy) and 75 IU of human menopausal gonadotropin (Menopur 75, Ferring Pharmaceuticals, West Drayton, UK). Ovulation was induced with recombinant human chorionic gonadotropin at a dose of 10,000 IU. EVs were obtained during oocyte retrieval after the selection of oocyte–cumulus complexes for ART programs. For the study, only residual FF EVs were used, which are normally discarded, have a transparent yellow color without admixture of blood and represent large fragments of granulosa cells. All study participants signed a voluntary informed consent form for the use of leftover EV samples for research purposes.

### 2.2. Isolation of Extracellular Vesicles from the Follicular Fluid by Differential Centrifugation

To isolate EVs from FF, the method of differential centrifugation was used. FF was selected without blood admixture using visual analysis. The collected FF in a volume of 5 mL was sequentially subjected to several stages of centrifugation to remove debris (400× *g* for 10 min, 10,000× *g* at 4 °C for 30 min). The supernatant was used to isolate the EVs by ultracentrifugation at 108,000× *g*. The resulting sediment was resuspended in 100 μL of PBS. The samples of EVs were stored at −80 °C.

### 2.3. Nanoparticle Tracking Analysis (NTA) of Extracellular Vesicles from Follicular Fluid

After the isolation of the FF EV sediment by differential centrifugation, the volume of the samples was adjusted to 100 μL with a PBS solution. Each sample was diluted immediately with PBS before measurement, according to the manufacturer’s instructions (Malvern, UK). The initial dilution factor used was 10. The samples (500–700 μL) were injected into a NanoSightLM10 instrument (Malvern Instruments, Malvern, UK) using a sterile 1 mL syringe. The capture and analysis settings were set manually according to the protocol. Using the NanoSight LM10 device, the FF EVs were visualized using laser light scattering, and the Brownian motion of the EVs was recorded on video. The number of tracks always exceeded 200, and the vesicle size and concentration were assessed for each sample. The recorded videos were then analyzed using NanoSight NTA 3.1 software (Malvern, UK). Individual particle scattering analysis for the detection of the diameter and concentration of the particles was carried out using 14 videos of 30 s for each sample at 25 °C.

### 2.4. Western Blotting 

Protein concentration was measured in EV samples using the Bradford method (Quick Star Bradford Protein Assay, Bio-Rad, Hercules, CA, USA, USA). EV markers were characterized by Western blotting. Each EV sample was mixed with 2× Laemmli sample buffer (Bio-rad, USA). The resulting EV samples were applied to the wells of a 10% PAAG gel in a volume of 10 μL (50 μg of protein). The proteins were separated at a field strength of 80 V in the concentrating gel and 180 V in the separating gel. This was followed by the semi-dry electrotransfer of proteins from the gel to a PVDF membrane using a Trans-Blot Turbo Transfer system (Bio-rad, USA). Following blocking, the membrane was incubated with the primary antibodies (Table S1) anti-C;D81 (Affinity Biosciences, DF8045, 1:1000), anti-CD63 (Affinity Biosciences, AF5117, 1:1000), anti-CD9 (Affinity Biosciences, AF5139, 1:1000) for 18 h at +4 °C overnight and then, after washing, with secondary antibodies—for 1 h at room temperature. The membrane was developed with an ECL substrate with signal detection in an automated ChemiScope instrument (Clinx, Shanghai, China).

### 2.5. Analysis of Lipids in the Extracellular Vesicles from Follicular Fluid Using Liquid Chromatography with Mass Spectrometry (LC-MS Analysis)

#### 2.5.1. Sample Preparation for the Mass Spectrometric Analysis of Lipid Extracts

The FF EV samples for the analysis of their lipid composition were obtained by pooling 15 samples from patients under 35 years of age and 15 samples from patients over 35 years of age. Lipid extracts were prepared according to the modified Folch method. TO this purpose, 480 μL of a chloroform/methanol solution (2:1, *v*/*v*) was added to an FF EV sample, and the mixture was incubated for 10 min, stirring thoroughly. Water was added to the resulting solution. The mixture was centrifuged at 13,000× *g* for 10 min at ambient temperature. Then, 150 µL of the organic lower layer containing lipids was taken, and 250 µL of the chloroform/methanol solution (2:1, *v*/*v*) was added to the remaining mixture, which was mixed again and centrifuged at 13,000× *g* for 10 min, after which, another 300 µL of the lower layer was taken. The organic phase was dried under a stream of nitrogen and then dissolved in 200 μL of acetonitrile/2-propanol (1:1, *v*/*v*) for subsequent mass spectrometric analysis.

#### 2.5.2. Mass Spectrometric Analysis of the Lipid Extracts

The lipid composition of the samples was determined by liquid chromatography–mass spectrometry on a Dionex UltiMate 3000 chromatograph (Thermo Scientific, Germany) coupled to a Maxis Impact qTOF mass analyzer with an elctrospray ion source (Bruker Daltonics, Bremen, Germany). The samples were separated by reversed-phase chromatography on a Zorbax C18 column (150 × 2.1 mm, 5 µm, Agilent, Santa Clara, CA, USA) with a gradient from 15% to 45% of eluent B over 2 min, and then from 45% to 99% of eluent B within 15 min. As eluent A, a solution of acetonitrile/water (60/40, *v*/*v*) with the addition of 0.1% formic acid and 10 mmol/L of ammonium formate was used. Eluent B was acetonitrile/isopropanol/water (90/8/2, *v*/*v*/*v*) supplemented with 0.1% formic acid and 10 mmol/L of ammonium formate. The elution flow rate was 35 μL/min, and the injected sample volume was 0.5 μL. Mass spectra were obtained in positive and negative ion mode in the *m*/*z* range of 100–1700 with the following settings: capillary voltage 4.1 kV for positive ion mode and 3.0 kV for negative ion mode, nebulizer gas pressure 0.7 bar, dry gas flow 6 L/min, and drying gas temperature 200 °C. For a detailed lipid identification, data-dependent tandem mass spectrometry was performed with an isolation window width of 5 Da.

#### 2.5.3. Lipid Identification

The msConvert from Proteowizard 3.0.9987 was used to convert files into the MzXml format, containing information about each mass spectrum at any point of time, and into the ms2 format, containing information about the tandem mass spectrum of an ion at a given time. MzMine was used for extracting peaks, normalizing to the total ion current and creating a table containing information about the peak, reporting *m*/*z* of the ion, peak area and retention time. Lipid identification was carried out by LipidMatch script [14]. Lipid nomenclature follows that in LIPID MAPS^®^.

### 2.6. Proteomic Analysis of Extracellular Vesicles from Follicular Fluid

#### 2.6.1. Sample Preparation for Proteomic Analysis of Extracellular Vesicles

The vesicle sediment was lysed in RIPA buffer, shaken for 30 min at 650 rpm, +4 °C, kept in ice water for 30 s and shaken again for 15 min. To reduce the –SH groups, DTT was added up to 10 mmol/L, and the resulting mixture was vortexed and incubated for 45 min at +50 °C. For alkylation, IAA was added up to 20 mmol/L; then, the resulting mixture was vortexed, drops were quickly discarded, and the samples were incubated (Eppendorf ThermoMixer, Hamburg, Germany) for 30 min in the dark at +25 °C. Then, 500 μL of ice-cold acetone was added to the samples, and the mixture was vortexed and left at −20 °C overnight. Before trypsinolysis, the samples were centrifuged (16,000× *g*, 10 min, +4 °C), and the supernatant was removed carefully, without touching the protein sediment. The protein pellet was resuspended in 50 μL of 40 mmol/L ABB, pH 8.0. Trypsin was added at a ratio of 1:50 to the protein mass. The mixture was incubated for 18 h at +37 °C and 350 rpm in a ThermoMixer. The samples were acidified with formic acid (FA), up to 1.5% (*v*/*v*) and pH = 2.0. The samples were centrifuged for 5 min at 16,000× *g*. When an insoluble precipitate formed, the supernatant was transferred to LoBind protein tubes.

#### 2.6.2. HPLC-MS/MS Analysis

The analysis of the peptide fraction was carried out on a Dionex Ultimate 3000 HPLC system (Thermo Fisher Scientific, Waltham, MA, USA) coupled to a TIMS TOF Pro mass spectrometer (Bruker Daltonics, Fremont, CA, USA), using the parallel accumulation and sequential fragmentation (PASEF) data acquisition method in DDA (data-dependent acquisition) mode. The electrospray source (ESI) settings were as follows: capillary voltage 4500 V, end-plate bias potential 500 V, and dry gas flow 3.0 L/min at 180 °C. The measurements were carried out in the mass/charge (*m/z*) range from 100 to 1700. The ion mobility range included values from 0.60 to 1.60 V s/cm^2^ (1/k_0_, where k_0_ is the ion mobility). The total cycle time was set to 1.16 s, and the number of PASEF MS/MS scans was 10. For small sample quantities, the total cycle time was set to 1.88 s.

For HPLC, the sample loading volume was 1 μL per injection. HPLC separation was performed using a packed emitter column (C18, 25 cm × 75 µm 1.6 µm, Ion Optics, Parkville, Australia) by the gradient elution method. Mobile phase A contained 0.1% formic acid in water; mobile phase B contained 0.1% formic acid in acetonitrile. LC separation was achieved at a flow rate of 400 nL/min in 40 min using a gradient from 4 to 90% of phase B.

#### 2.6.3. Protein Identification

The obtained data were analyzed using PEAKS Studio 8.5 software, using the following parameters: parent ion mass measurement error, 0 ppm; and fragment mass error, 0.03 Da. Methionine oxidation was identified as a possible variable modification. The search was performed using the Swissprot database of human proteins. The FDR thresholds for all stages were set at 0.01 (1%) or lower.

### 2.7. Evaluation of Progesterone in EVs

#### 2.7.1. Sample Preparation for the Evaluation of Progesterone

For this analysis, 400 μL of methanol was added to the EV sample placed in a 1.5 mL Eppenorf tube, mixed using a multivortex for 5 min and centrifuged at 4 °C for 10 min at 15,000 rpm. Then, 300 µL of the supernatant was transferred into a clean 2 mL Eppendorf tube, and 1000 µL of tert-methylbutyl ether was added, followed by mixing using a multivortex for 10 min and centrifugation at 4 °C for 10 min at 15,000 rpm. At this point, 750 µL of the supernatant was collected in a clean 2 mL Eppendorf tube and then another 1000 µL of tert-methylbutyl ether was added. After mixing using a multivortex for 5 min, centrifugation was performed at 4 °C for 10 min at 15,000 rpm. Then, 1000 μL of the supernatant was collected and dried using a concentrator under a stream of nitrogen at 40 °C. The dry residue was redissolved in a solution of water/acetonitrile/formic acid (90/10/0.1, *v/v/v*), mixed using a multivortex for 5 min and centrifuged at 4 °C for 10 min at 15,000 rpm. Finally, 70 μL of the supernatant was transferred into a vial with an insert. The injection volume was 50 µL.

#### 2.7.2. HPLC-MS/MS Analysis of Progesterone

Chromatographic separation was carried out on an Agilent Technologies 1260 Infinity system with mass spectrometric detection (QTRAP 5500 ABSciex, Toronto, ON, Canada) in electrospray mode. Mobile phase A was a 0.1% solution of formic acid in water, mobile phase B was a 0.1% solution of formic acid in acetonitrile. The samples were separated by reversed-phase chromatography on a Poroshell 120 EC-C18 column (100 × 2.1 mm, 2.7 µm, Agilent, USA) with a gradient from 10% to 35% of eluent B over 0.1 min, from 35% to 65% of eluent B over 10.3 min and then from 65% to 95% of eluent B over 1.5 min at a flow rate of 550 µL/min and a column temperature of 50 °C. Mass spectra were obtained in positive ion multiple reaction monitoring mode with *m/z* 315.2/97.0 and 315.2/109.1 transitions with the following settings: declustering potential 111 V, collision energy 25 and 31 for the first and the second transition, respectively, curtain gas 20 psi, gas1 45 psi, gas2 45 psi, and ion spray voltage 5500 V.

### 2.8. Statistical Analysis

MS Excel 2008 (Microsoft Corporation Redmond, Washington, DC, USA) and STATISTICA6 (StatSoft Inc., Palo Alto, CA, USA) were used for the statistical analysis and the diagrams. The normality of the distribution was tested using the Shapiro–Wilk test. All values are expressed as mean ± standard error (SEM). The statistical analyses were performed by paired *t*-tests.

## 3. Results

### 3.1. Characterization of Extracellular Vesicles

**Figure 1:**
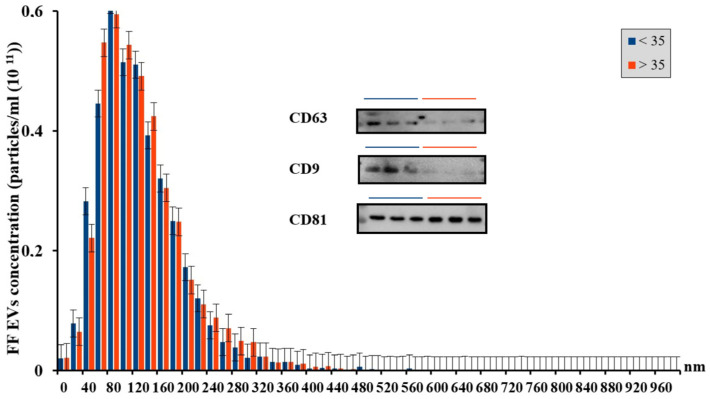
Histogram displaying the size distribution and concentration of FF EVs from women of young (blue) and old (red) reproductive age. The data were obtained using nanoparticle tracking analysis. Shown are the means ± SEM. Detection of extracellular vesicle markers, including CD63, CD9, and CD81, using Western blot analysis.

For isolation of EVs from the FF, the method of differential centrifugation was used, which made it possible to conduct the NTA. Since age is an important parameter affecting women reproductive function, follicular fluid was stratified into two groups: from young patients under 35 years old and from advanced-maternal-age (over 36 years of age) patients. The concentration and hydrodynamic size of particles in the FF from women of different reproductive ages were assessed. The data are presented in Figure 1.

NTA showed that there were no significant differences in vesicle concentration and size between the age groups: for the group of women <35 years old, the concentration was (3.75 ± 0.47) × 10^11^ parts/mL, SD = 0.8, *n* = 15, for the group of women >35 years old, the concentration was (3.7 ± 0.6) × 10^11^ parts/mL, SD = 1.036, *n* = 14, and the average particle size was 138.78 ± 9.92 nm, SD = 17.19 and 127.03 ± 17.17 nm, SD = 29.7, respectively. For both age groups, there was a large number of FF EVs of small and medium size, from 40 to 200 nm, which correspond to microvesicles and exosomes, in agreement with literature data [4]. However, for the group of advanced maternal age, there was a tendency towards an increase in the number of large-sized FF EVs, presumably apoptotic bodies with sizes between 200 and 300 nm.

### 3.2. Assessment of the Progesterone Levels in Extracellular Vesicle Samples from Female Follicular Fluid

**Figure 2:**
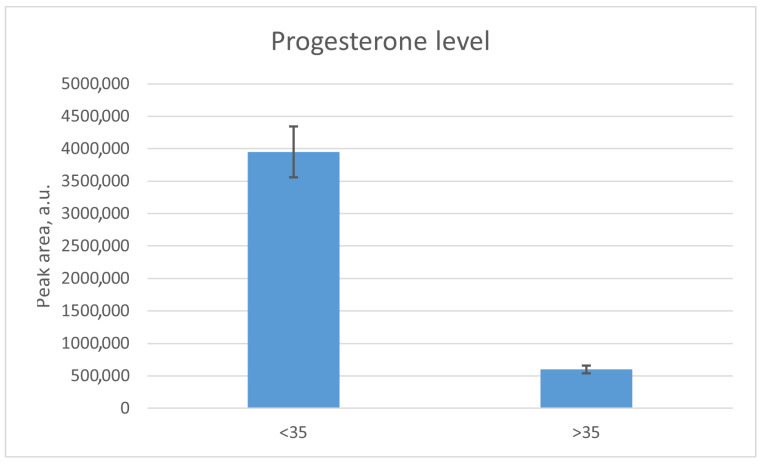
Progesterone levels in the samples of EVs from patients under and over 35 years old. Bars represent measurement uncertainty calculated as the standard deviation of triplicate analyses of the same sample.

**Figure 3:**
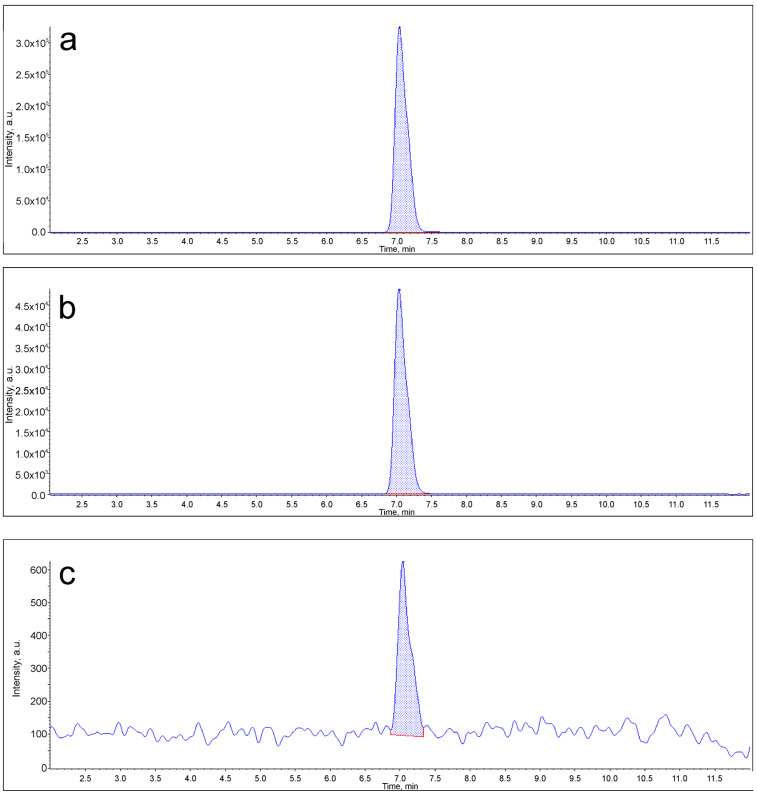
Chromatogram of the ion current of the *m/z* 315.2/97.0 transition, corresponding to progesterone. (**a**) Sample of vesicles from patients under 35 years of age; (**b**) sample of vesicles from patients over 35 years old; (**c**) blank sample. The blue line indicates the extracted ion current level; the red line shows the noise level used to calculate the chromatographic peak area.

As a result of the HPLC-MS/MS analysis of the EV samples, it was revealed that the level of progesterone in the vesicles from patients under 35 years old (chromatographic peak area 3,951,000 arbitrary units) was 6.6 times higher than the level of progesterone in the vesicles from patients over 35 years old (chromatographic peak area 598,500 arbitrary units) (*p* < 0.05, Figure 2 and Figure 3).

### 3.3. Lipidomic Analysis of Extracellular Vesicles from the Follicular Fluid of Women of Different Reproductive Ages

As a result of the lipidomic analysis, 389 lipids were identified (Supplementary Materials, Table S2), of which 74 lipids were identified by exact mass and characteristic fragments in their tandem mass spectra, and 315 lipids by exact mass only. In total, 23 classes were identified: cholesteryl esters (CEs), cholesterols, N-lignoceroylsphingosines (Cer-NSs), lysophosphatidylcholines (LPC)s, phosphatidylcholines (PCs), phosphatidylethanols (PEtOHs), ethers of triglycerides and phosphatidylcholines (plasmanyl-TGs, plasmenyl-PCs), sphingomyelins (SMs), triglycerides (TGs), phosphatidic acids (PAs), acylcarnitines (AcCas), ceramides (Cers), glycosylceramides (CerGs), ceramide phosphates (CerPs), coenzymes (Cos), diglycerides (DGs), monoglycerides (MGs), monogalactosyldiacylglycerols (MGDG)s, oxidized forms of lysophosphatidylcholines, phosphatidylcholines, phosphatidylethanolamines and triglycerides (OxLPCs, OxPCs, OxPEs, OxTGs).

**Figure 4:**
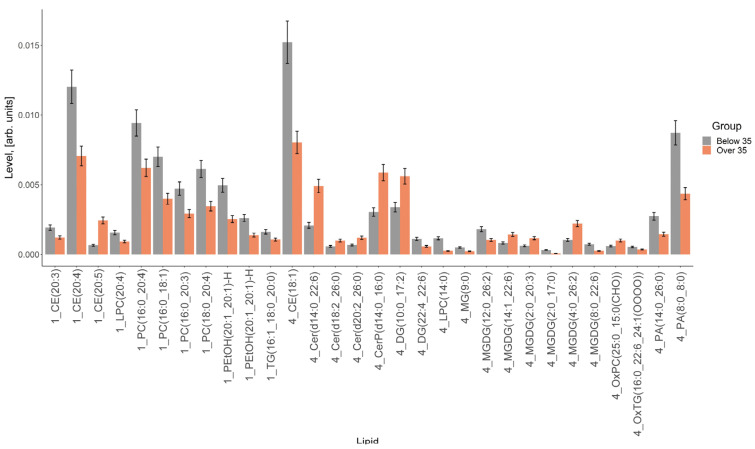
Comparison of lipids, the levels of which differed between the groups by more than 1.5 times. Bars represent measurement uncertainty calculated as the standard deviation of triplicate analyses of the same sample.

The result of the comparison of the relative lipid levels between the study groups is presented in Figure 4, which shows lipids whose levels differed by more than 1.5 times. The greatest differences between the groups were found for individual cholesterol esters, lysophosphatidylcholines, phosphatidylcholines, ceramides, triglycerides, di- and triglycerides and monogalactosyldiacylglycerols (Figure 4).

**Figure 5:**
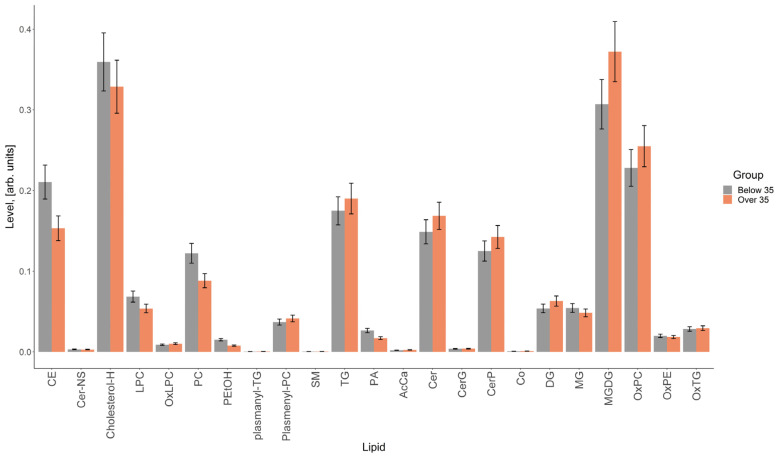
Comparison of total lipid levels for each identified class. CE—cholesteryl esters, Cer-NS—cholesterols, N-lignoceroylsphingosines, LPC—lysophosphatidylcholines, PC—phosphatidylcholines, PEtOH—phosphatidylethanols, plasmanyl-TG, plasmenyl-PC—ethers of triglycerides and phosphatidylcholines, SM—sphingomyelins, TG—triglycerides, PA—phosphatidic acids, AcCa—acylcarnitines, Cer—ceramides, CerG—glycosylceramides, CerP—ceramide phosphates, Co—coenzyme, DG—diglycerides, MG—monoglycerides, MGDG—monogalactosyldiacylglycerols, OxLPC, OxPC, OxPE, OxTG—oxidized forms of lysophosphatidylcholines, phosphatidylcholines, phosphatidylethanolamines and triglycerides. Bars represent measurement uncertainty calculated as the standard deviation of triplicate analyses of the same sample.

The total levels of cholesteryl esters, cholesterol, lysophosphatidylcholines and phosphatidylcholines were higher in the vesicles obtained from women under 35 years of age, and those of di- and triglycerides, monogalactosyldiacylglycerols, oxidized forms of phosphatidylcholines and ceramides were higher in the vesicles obtained from women over 35 years old (Figure 5).

### 3.4. Proteome of Extracellular Vesicles from Follicular Fluid 

In this study, untargeted proteomics analysis was carried out by HPLC-MS/MS analysis to characterize the proteome of human FF EVs. About 1000 proteins were identified, including both EV marker proteins and those specific for fertilization processes. The participation of the latter in the activation of human sperm in the fallopian tube is presented in this work for the first time. 

The identified proteins belong to various functional categories, including growth factors and hormones, receptor signaling, enzyme catalysis, etc. [15]. This work can serve as a resource for future research aimed at developing biomarkers to determine not only the quality of oocytes but also their reproductive potential, as well as at studying the processes of interaction between oocyte and sperm and their changes in morphofunctional characteristics.

Table 1 presents the main proteins characteristic of EVs. CD9, CD81, CD63 are tetraspanins that play a role in cell entry, invasion and fusion. They are also used as exosome markers. Another type of exosome proteins consists of heat shock proteins such as HSP70, HSP90, which are part of the stress response and are involved in antigen presentation. Cytoskeletal proteins (e.g., tubulin, actin) and proteins responsible for membrane transport, including annexins (A6, A5, A2, A11, A4), were also detected and identified in EV exosomes. In turn, proteins such as flotillin and TSG101 are involved in the biogenesis of exosomes [15,16]. It was shown that FF EVs contain metabolic enzymes such as ATPase, aldehyde reductase and aspartate aminotransferase, which may be involved in the activation of sperm in the reproductive tract, increasing their motility and triggering hyperactivation.

An experiment to determine proteins in the FF EVs showed the presence of specific proteins that may be candidates for factors that trigger the processes of capacitation and hyperactivation of sperm in the fallopian tubes, thereby preparing sperm for fertilization and enhancing the selection of the most competent sperm (Table 2). These are proteins involved in the binding and transfer of Ca^2+^ ions, which is necessary for sperm to improve motility, phospholipases, which trigger the acrosomal reaction, and G proteins and associated factors, necessary to trigger the work of Ca^2+^ channels, activate capacitation and bind to the oocyte CP. A membrane receptor for progesterone was also discovered, which, as the results of the work showed, was present in greater quantities on the EVs from young women and thus has a great influence on the activation of sperm.

Of interest was the rather large percentage of coverage in the samples of the Tsukushi protein, which is massively expressed in the female genital tract and is directly involved in the regulation of the activity of G proteins.

## 4. Discussion

The human FF is one of the most complex biological fluids. It not only serves as a microenvironment for the follicles in the ovary and supports the development of the egg cells, but also regulates the fertilization processes in the fallopian tube. This regulation includes the attraction of the sperm to the oocyte and its activation and preparation for penetration in the egg, which is facilitated by signaling proteins and EVs of the reproductive system. Proteins and other bioactive molecules are transported by EVs, and insights into the protein composition of FF EVs could improve our understanding of ovarian physiology and oocyte development and competence and open new avenues for studying the disruption of molecular processes during fertilization. These data may help to understand the causes of unexplained infertility in ART programs. However, the composition of the FF EV proteome remains poorly understood, and one of the main difficulties remains the identification of proteins specific to FF EVs that interact with spermatozoa and influence their functional properties.

Cells secrete EVs of various sizes and intracellular origin. Microvesicles (also known as ectosomes) are formed from the plasma membrane by budding [17], while exosomes are formed from multivesicular bodies (MVBs) and are released in well-organized systems. Exosomal cargos, such as proteins, lipids and nucleic acids, are selectively incorporated into intraluminal vesicles (ILVs), i.e., precursors of exosomes, in MVBs. MVBs are then transported to the plasma membrane, and after fusion with it, MVBs are released into the extracellular space in the form of exosomes [18]. The results of our Western blot analysis showed that three markers (CD63, CD81 and CD9) were detected in EVs obtained from follicular fluid, and two of them (CD63, CD9) seemed to be down-regulated in the group of higher age. Silveira and colleagues revealed the positive effect of CD63+ exosomal vesicles isolated from follicular fluid on the proper development of mammalian embryos, in particular cattle [19]. Another study using cytometry showed that, with age, there is a decrease in the total number of CD63+ markers in urine [20]. It was also experimentally revealed that with age, in rats, there was an increase in the level of CD63 in exosomes from the cerebrospinal fluid and a decrease in CD63 in plasma vesicles in the older group [21]. CD9, in turn, influences the interaction between sperm and oocyte, as well as embryo implantation in humans [22]. In a CD9/CD81 double-knockout mouse model, a syndrome reminiscent of human aging gradually developed, leading to atrophy of various organs [23]. These data may indicate a change in CD9+ concentration in the follicular fluid. It should be noted that the concentration of exosomes and exosomal proteins in the FF from an aged group of women was slightly lower than that in the FF from a group of young women [24].

In this study, we detected an increase in the number of large EVs in the FF of women of advanced reproductive age, and there were no significant differences in EV concentration and size between the age groups. We assume that this was due to the fact that the FF for EV isolation was selected only from the dominant ovulating follicle in all women of young and older reproductive age. However, conflicting results are found in the literature. These studies were carried out on different models, using different sources of EVs and isolation methods, which indicates the need for further research. Thus, in a study by Rosalia Battaglia et al., there was a greater amount of EVs in the follicular fluid of women of late reproductive age compared to that of younger ones; the same was observed in a study by Matilde Alique et al. in human plasma [25,26]. Erez Eitan et al. found that the concentration of EVs in blood plasma decreased with age [27]. Comparing the sizes of EVs, a study by Zhang et al. found that EVs isolated from the blood serum of elderly rats had a larger average diameter compared to those from young ones [15], and Rosalia Battaglia et al. found a higher concentration of small EVs in the FF of women of late reproductive age (1.1 × 10^11^ EV/mL) compared to that of young women (4.5 × 10 EV/mL) [27]. 

It is known that one cell can secrete different types of EVs (in size and/or composition) [15]. Although several different mechanisms of exosome biogenesis have been reported [16,18], how these mechanisms are differentially used or regulated within a single cell remains completely unknown. This is mainly due to the fact that the research results vary depending on the methods and devices used to obtain them. Thus, the mechanisms by which heterogeneous populations of EVs are formed have not been sufficiently studied, and our work puts forward the hypothesis that the active participation of EVs in many processes, including in the reproductive system, depends to a greater extent, rather than on their size, on their molecular composition, varying depending on pathological and age-related changes.

The involvement of EVs in the aging process has been demonstrated in a variety of cell models, including humans [28]. Although the most well-known components of the SASP (aging-associated secretory phenotype) are soluble factors such as pro-inflammatory interleukins, growth factors and chemokines, much attention is currently being paid to the study of EVs, which are considered to be one of the key participants in SASP [29].

As is known, age-related changes primarily affect the reproductive system [11,25], and the composition of FF EVs may indicate not only changes in maintaining the functioning of the follicles, but also a deterioration in the processes of interaction between oocyte and sperm during natural fertilization.

The results presented in this work indicate a trend according to which the level of vesicular progesterone from the FF EVs was notably higher (6.6-fold) in women under 35 years of age. Numerous studies suggested that progesterone in FF EVs plays a key role in attracting sperm to the oocyte and in their hyperactivation and acquisition of fertilizing ability [30,31,32], activating the Ca^2+^ dependent channel CatSper; our previous work also confirms these results [1]. Progesterone is the main signal for the release of sperm from the isthmus of the oviduct, known as the functional sperm reservoir, and is the main chemoattractant in humans [33]. Since the hormonal and molecular composition of FF EVs changes with age, the amount of progesterone carried by FF EVs may also decrease significantly as women approach menopause, which can significantly affect the processes of sperm activation in the reproductive tract and their fertilizing ability.

The biochemical composition of the EVs of young women and of women of older reproductive age also differed in proteins and lipids. The results obtained on the lipid composition of FF EVs made it possible to determine that the total levels of cholesteryl esters, cholesterol, lysophosphatidylcholines and phosphatidylcholines were higher in the vesicles obtained from women under 35 years of age, and those of di- and triglycerides, monogalactosyldiacylglycerols, oxidized forms of phosphatidylcholines and ceramides were higher in the vesicles received from women over 35 years of age. In a study by Khayrullin et al., a lipidomic analysis of serum extracellular vesicles revealed that the serum EVs of older women were highly enriched in ceramide and were significantly different from those of younger women [34]. Very long chain lipotoxic ceramides can cause mitochondrial dysfunction, oxidative stress and cell death in cardiomyocytes [5]. Thus, EV-derived ceramide represents a novel factor in the aging process that may contribute to degeneration in many organs and tissues [34]. Therefore, the lipidomic analysis of EVs can also be used to study the role of EVs in the aging process. Ceramide is a central component of sphingolipid metabolism and is involved in the synthesis of other interrelated bioactive lipids, controlling cellular reactions and participating in the regulation of many physiological functions, including exocytosis and, in particular, the acrosomal reaction of human spermatozoa [35]. Ceramides can be formed de novo as a result of sphingomyelin hydrolysis, but such a process was not found in sperm. We assume that ceramide enters the sperm and activates their fertilizing function. Among the participants in the process, ceramide from FF EVs binds to sperm membrane receptors and triggers the necessary processes supporting fertilization. However, the increased level of oxidized ceramides in the SRV group indicated the presence of less functional molecules in these IVF samples, since lipid oxidation is associated with pathological conditions of the body, oxidative stress, and dysfunctional metabolic changes [36,37].

Elevated levels of cholesterol and cholesteryl esters support the hypothesis that age-related metabolic changes influence both the quality of the oocyte and its ability to attract and activate sperm. Cholesterol is a participant in many regulatory and energy processes and is a substrate in the preparation and process of fertilization. With age, the severity and success of these events decrease, as does a person’s reproductive potential.

In this study, about 1000 proteins were identified, both markers of and specific for fertilization processes. This is the first study to demonstrate the involvement of specific EV proteins in the activation of human sperm in the fallopian tube.

c-AMP is one of many molecules responsible for the processes of capacitation and hyperactivation, which is transferred by FF EVs into the cavity of the fallopian tube during ovulation. In 2017, Alonso et al. showed that different concentrations of c-AMP induced sperm capacitation, increasing sperm Ca^2+^ levels (*p* < 0.01) through the activation of different signaling pathways. In addition, c-AMP-induced sperm hyperactivation through the interaction with EVs contributed to an increase in the proportion of sperm with high mitochondrial activity (*p* < 0.01). Finally, c-AMP increased the rate of in vitro fertilization compared with control conditions (*p* < 0.001) [38].

The annexin protein, being one of the EV markers, can also perform a large number of functions, changing the morphofunctional characteristics of human sperm. In animal models, it was shown that annexin A2 (ANXA2) regulated miRNA loading in EVs in a sequence-independent manner and bound miRNAs in EVs in the presence of Ca^2+^ and that insufficient ANXA2 reduced the amount of miRNAs in EVs, which might be a characteristic of increasing age [39]. It was also shown in various mammalian species (mice, hamsters, bulls and pigs) that annexin 1, 2, 4, 5 are involved in the retention of sperm in the fallopian tube reservoir until ovulation and in changes in the sperm membrane at the beginning of capacitation [39,40].

Various types of phospholipases are involved in the processes of exocytosis in the mammalian body, including the acrosomal reaction of sperm. The significance of the presence of phospholipase D in the transition of hyperactivated sperm to capacitation and in further preparation of the membrane for fertilization was experimentally demonstrated in rats, rabbits, bulls and humans in vitro [41,42].

Thanks to the proteomic analysis of EVs in this work, a unique protein, Tsukushi (TSK), was discovered that regulates the activity of G protein-coupled receptors and is most strongly expressed in the reproductive system. Recently discovered, TSK regulates signaling pathways that ultimately control proliferation and cellular communication. In recent years, research on TSK has become increasingly sophisticated, illustrating its involvement in the physiology and pathophysiology of neuronal, genetic and metabolic diseases [43]. There are currently no publications on the study of TSK in fertilization processes. This work may develop interest in further research in this direction, since the indirect participation of TSK in the processes of sperm activation was determined by its localization and molecular functions. Furthermore, according to the available literature, the specific proteins listed in Table 2 not only have the ability to improve the morphofunctional properties of spermatozoa in different species, but also may act as therapeutic agents. Their biological activities include the regulation of G-protein-coupled receptors and the promotion of sperm fertilization capacity by increasing ion levels via different signaling pathways. In addition, these proteins play a crucial role in altering the sperm membrane, which underlines their pivotal role in the reproductive process.

## 5. Conclusions

Our results highlight the complexity and multifaceted nature of the factors controlling the fertilization process and reveal numerous elements that remain to be clarified. We found that EVs play a crucial role not only in oogenesis, implantation and embryogenesis, but also in improving the functional parameters of spermatozoa through EV-mediated interactions. This discovery provides invaluable insights into the fundamental mechanisms of gamete cell interaction, including the selective process by which oocytes associate with specific sperm for fertilization, thereby expanding our understanding of reproductive physiology. In addition, the application of IVF technologies offers the dual benefit of deepening our understanding of human reproductive biology and improving the clinical outcomes of ART programs. Our research points to the particularly promising strategy of using donor extracellular vesicles from the follicular fluid of healthy, young women to potentially ‘rejuvenate’ gametes in patients of advanced reproductive age.

## Figures and Tables

**Table 1:** Markers and characteristic proteins of FF EVs.

Affiliation, Name	Coverage, %	Description
P08133|ANXA6_HUMAN	42	Annexin A6
P21926|CD9_ HUMAN	21	CD9 antigen OS = Homo sapiens OX = 9606 GN = CD9 PE = 1 SV = 4
P60033|CD81_ HUMAN	25	CD81 antigen OS = Homo sapiens OX = 9606 GN = CD81 PE = 1 SV = 1
O43657|TSN6_ HUMAN	16	Tetraspanin-6 OS = Homo sapiens OX = 9606 GN = TSPAN6 PE = 1 SV = 1
P08758|ANXA5_HUMAN	27	Annexin A5 OS = Homo sapiens OX = 9606 GN = ANXA5 PE = 1 SV = 2
P07355|ANXA2_HUMAN	14	Annexin A2 OS = Homo sapiens OX = 9606 GN = ANXA2 PE = 1 SV = 2
P50995|ANX11_HUMAN	15	Annexin A11 OS = Homo sapiens OX = 9606 GN = ANXA11 PE = 1 SV = 1
P09525|ANXA4_HUMAN	7	Annexin A4 OS = Homo sapiens OX = 9606 GN = ANXA4 PE = 1 SV = 4
O75955|FLOT1_HUMAN	11	Flotillin-1 OS = Homo sapiens OX = 9606 GN = FLOT1 PE = 1 SV = 3
P08962|CD63_ HUMAN	5	CD63 antigen OS = Homo sapiens OX = 9606 GN = CD63 PE = 1 SV = 2
O75954|TSN9_ HUMAN	6	Tetraspanin-9 OS = Homo sapiens OX = 9606 GN = TSPAN9 PE = 1 SV = 1
P20073|ANXA7_HUMAN	3	Annexin A7 OS = Homo sapiens OX = 9606 GN = ANXA7 PE = 1 SV = 3
P16070|CD44_ HUMAN	3	CD44 antigen OS = Homo sapiens OX = 9606 GN = CD44 PE = 1 SV = 3
P04083|ANXA1_HUMAN	13	Annexin A1 OS = Homo sapiens OX = 9606 GN = ANXA1 PE = 1 SV = 2
P41732|TSN7_ HUMAN	6	Tetraspanin-7 OS = Homo sapiens OX = 9606 GN = TSPAN7 PE = 1 SV = 2
Q99816|TS101_ HUMAN	4	Tumor susceptibility gene 101 protein OS = Homo sapiens OX = 9606 GN = TSG101 PE = 1 SV = 2
P0DMV8|HS71A_HUMAN	4	Heat shock 70 kDa protein 1A OS = Homo sapiens OX = 9606 GN = HSPA1A PE = 1 SV = 1
P0DMV9|HS71B_HUMAN	4	Heat shock 70 kDa protein 1B OS = Homo sapiens OX = 9606 GN = HSPA1B PE = 1 SV = 1

**Table 2:** Specific FF EV proteins presumably involved in the activation of capacitation, hyperactivation and preparation of sperm for fertilization in the female reproductive tract.

Affiliation, Name	Coverage, %	Description
P23634|AT2B4_HUMAN	17	Plasma membrane calcium-transporting ATPase 4 OS = Homo sapiens OX = 9606 GN = ATP2B4 PE = 1 SV = 2
Q16610|ECM1_HUMAN	47	Extracellular matrix protein 1 OS = Homo sapiens OX = 9606 GN = ECM1 PE = 1 SV = 2
Q08380|LG3BP_HUMAN	51	Galectin-3-binding protein OS = Homo sapiens OX = 9606 GN = LGALS3BP PE = 1 SV = 1
P00558|PGK1_HUMAN	13	Phosphoglycerate kinase 1 OS = Homo sapiens OX = 9606 GN = PGK1 PE = 1 SV = 3
P78527|PRKDC_HUMAN	11	DNA-dependent protein kinase catalytic subunit OS = Homo sapiens OX = 9606 GN = PRKDC PE = 1 SV = 3
P54289|CA2D1_HUMAN	6	Voltage-dependent calcium channel subunit alpha-2/delta-1 OS = Homo sapiens OX = 9606 GN = CACNA2D1 PE = 1 SV = 3
Q8WUA8|TSK_HUMAN	29	Tsukushi OS = Homo sapiens OX = 9606 GN = TSKU PE = 1 SV = 3
Q9Y653|AGRG1_HUMAN	6	Adhesion G-protein-coupled receptor G1 OS = Homo sapiens OX = 9606 GN = ADGRG1 PE = 1 SV = 2
P31323|KAP3_HUMAN	5	cAMP-dependent protein kinase type II-beta regulatory subunit OS = Homo sapiens OX = 9606 GN = PRKAR2B PE = 1 SV = 3
P80108|PHLD_HUMAN	27	Phosphatidylinositol-glycan-specific phospholipase D OS = Homo sapiens OX = 9606 GN = GPLD1 PE = 1 SV = 3
O15173|PGRC2_HUMAN	9	Membrane-associated progesterone receptor component 2 OS = Homo sapiens OX = 9606 GN = PGRMC2 PE = 1 SV = 1
P17301|ITA2_HUMAN	17	Integrin alpha-2 OS = Homo sapiens OX = 9606 GN = ITGA2 PE = 1 SV = 1
P05556|ITB1_HUMAN	12	Integrin beta-1 OS = Homo sapiens OX = 9606 GN = ITGB1 PE = 1 SV = 2

## Data Availability

Data supporting the results presented in this article are available upon request to the authors.

## Supplementary Materials

The supporting information can be downloaded at https://www.mdpi.com/article/10.3390/life14050541/s1. Table S1: Antibodies which were used for western blotting for EVs characterization. Table S2: Result of lipid identification in follicular fluid extracellular vesicle samples.
