# Supplementary material for: Characteristics of the Follicular Fluid Extracellular Vesicle Molecular Profile in Women in Different Age Groups in ART Programs
Source: Life (Basel). 2024 Apr 24;14(5):541. doi: 10.3390/life14050541 (PMC11121889; doi:10.3390/life14050541)
Supplement: Supplementary file 1 [file life-14-00541-s001.zip › life-2920509-supplementary Table S1.pdf]

Table S1. Antibodies which were used for western blotting for EVs characterization.

Name	Catalog number	Organism	Application	Manufacturer	Concentrations
CD81 Antibody	DF8045	Human	Western blot; enzyme immunoassay	Affinity Biosciences, China	1:1000
CD63 Antibody	AF5117	Human, Mouse, Rat	Western blot; immunohistochemistry, enzyme immunoassay	Affinity Biosciences, China	1:1000
CD9 Antibody	AF5139	Human, Mouse, Rat	Western blot; immunohistochemistry, immunofluorescence/immunocytochemistry, enzyme immunoassay	Affinity Biosciences, China	1:1000
